# Endoscopic submucosal dissection with a double-endoscope technique for treating superficial esophageal neoplasms

**DOI:** 10.1055/a-2897-7597

**Published:** 2026-08-11

**Authors:** Jiankun Wang, Xiaohan Jiang, Li Liu

**Affiliations:** 1 Department of Geriatric Gastroenterology 74734 The First Affiliated Hospital with Nanjing Medical University & Jiangsu Province HospitalNanjingJiangsuChina

**Fig. 1:**
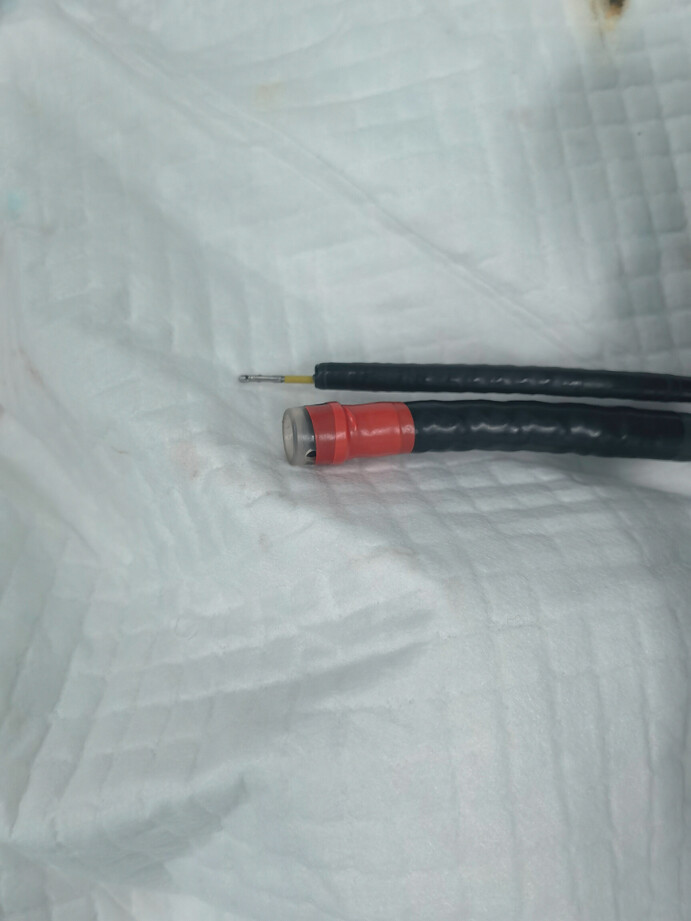
The ultrathin gastroscope and ultrathin biopsy forceps used in the ESD procedure.

Endoscopic submucosal dissection (ESD) of the esophagus remains one of the most challenging endoscopic procedures, particularly for large lesions. Effective traction can facilitate adequate exposure of the submucosal layer, thereby reducing the difficulty of ESD. 1​ In recent years, double-endoscope techniques have been successfully applied for gastric and colorectal lesions 2​ 3​ ​4​ ; however, their use in the esophagus has not yet been reported. Here, we describe a case in which a double-endoscope approach was employed to rapidly and efficiently perform ESD for a large esophageal lesion. By using an ultrathin gastroscope (Olympus, Tokyo, Japan) in combination with an ultrathin biopsy forceps (Micro-Tech, Nanjing, China) to adjust the traction direction and force ( Fig. 1 ), the lesion was ultimately resected with a standard gastroscope (Olympus, Tokyo, Japan).

**Fig. 2:**
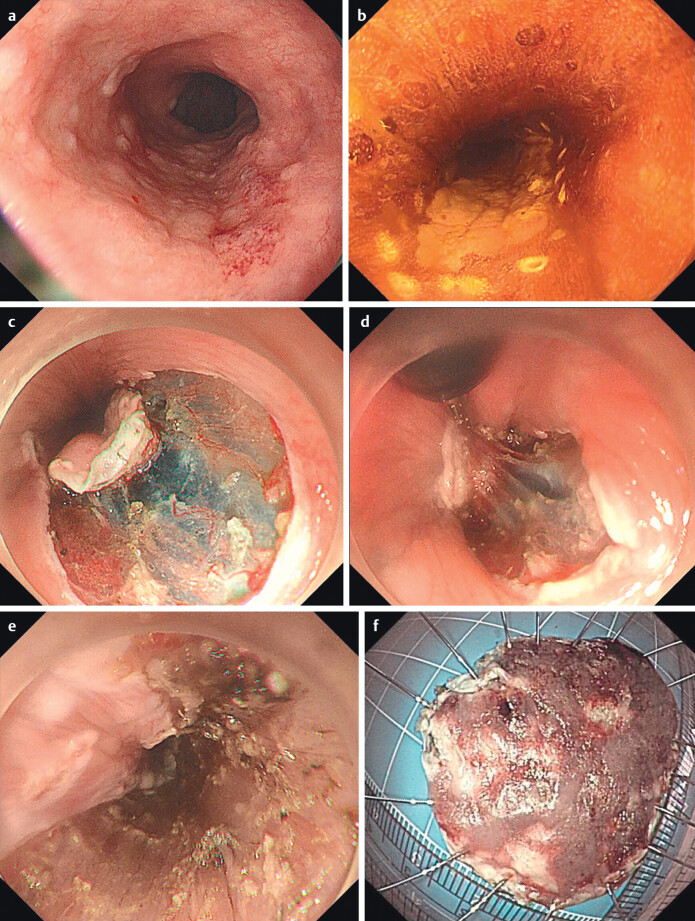
( **a** ) Mid-esophageal lesion. ( **b** ) Circumferential marking around the lesion. ( **c** ) Mucosal incision. ( **d** ) Ultrathin biopsy forceps grasping the edge of the lesion to provide traction. ( **e** ) Post-ESD mucosal defect. ( **f** ) Resected specimen.

We report the case of a 73-year-old man with a mid-esophageal lesion. Magnifying endoscopy and narrow-band imaging assessment indicated that the lesion was suitable for ESD. Due to the large lesion size and anticipated technical difficulty ( Fig. 2a ), we opted for a double-endoscope approach. As shown in Video 1 , after marking the lesion ( Fig. 2b ), a submucosal injection was performed to lift the lesion. Mucosal incision was then carried out using a DualKnife ( Fig. 2c ). Given the narrow esophageal lumen, an ultrathin gastroscope was employed to assist with traction. Under endoscopic visualization, the ultrathin gastroscope was advanced to the vicinity of the lesion, and an ultrathin biopsy forceps was introduced through its working channel. During submucosal dissection, the biopsy forceps was used to grasp the mucosal edge ( Fig. 2d ), while the ultrathin gastroscope continuously adjusted the direction to provide effective traction, thereby fully exposing the submucosal layer and improving dissection efficiency. As the procedure progressed, the biopsy forceps could be repositioned to grasp different parts of the mucosa under the control of the ultrathin gastroscope to further optimize submucosal exposure. Ultimately, ESD was completed efficiently and safely, requiring only 21 minutes, with no adverse events ( Fig. 2e, f ). Histopathology confirmed negative margins, achieving R0 resection.

**Video 1** Double-endoscope assisted endoscopic submucosal dissection with ultrathin biopsy forceps for large esophageal lesions.


In summary, the combination of an ultrathin gastroscope with a standard gastroscope offers significant facilitation for esophageal ESD without substantial additional cost, and may be considered for broader clinical application.

Endoscopy_UCTN_Code_TTT_1AO_2AC
